# Molecularly Imprinting Polymers (MIP) Based on Nitrogen Doped Carbon Dots and MIL-101(Fe) for Doxorubicin Hydrochloride Delivery

**DOI:** 10.3390/nano10091655

**Published:** 2020-08-23

**Authors:** Yuqiong Shi, Yuxuan Wang, Jinhua Zhu, Wei Liu, Md. Zaved H. Khan, Xiuhua Liu

**Affiliations:** 1 Henan International Joint Laboratory of Medicinal Plants Utilization, College of Chemistry and Chemical Engineering, Henan University, Kaifeng 475004, China; sq13027537872@163.com (Y.S.); wyx15224211156@163.com (Y.W.); 15290809123@163.com (W.L.); 2 Department of Chemical Engineering, Jessore University of Science and Technology, Jessore 7408, Bangladesh; zaved.khan@yahoo.com

**Keywords:** MIP, nanocomposites, drug delivery, DOX, controlled release

## Abstract

MIL-based molecularly imprinted polymer (MIP) nanocomposites were successfully synthesized through a simple and versatile stirring auxiliary encapsulation method. MIP as a carrier has been applied to the highly efficient selective recognition and sustained release of doxorubicin hydrochloride (DOX). The adsorption mechanism and release behavior of MIP@DOX in vitro were also discussed. Adsorption studies showed that MIP using DOX as template had specific selectivity to DOX, and its optimal drug loading efficiency reached 97.99%. The adsorption isotherm accorded with Freundlich models. The cumulative release curve showed that at the conditions of pH 5.5 and 7.4, the nanomaterials have a slow-release effect on the release of DOX. In addition, the cytotoxicity and bioactivity of MIP nanoparticles on HepG2 and HL-7702 cell lines measured by MTT assay also proved their low toxicity and biological activity. The cell activity of HepG2 and HL-7702 incubated with MIP for 24 h was 69.9% and 76.07%, respectively. These results collectively illustrated that the MIP nano-materials synthesized in this study can be efficiently employed to the drug delivery systems.

## 1. Introduction

Cancer is a main disease that jeopardizes human health and poses a critical threat to human life, and the incidence and mortality of cancer have been on the rise in recent years [1,2,3]. Doxorubicin hydrochloride (DOX) is an effective anticancer drug, which can remarkably inhibit the growth of cancer cells [4]. However, due to its toxicity to normal tissues and its inherent multidrug resistance effect, it is less appealing for direct application [5]. Therefore, many researchers are endeavoring to research and develop drug delivery systems (DDS) for DOX. For this purpose, polymer conjugates [6,7], metal-organic frameworks [8,9,10], and polymeric micelles [11] have been applied as carriers for DOX delivery.

Although the above carriers play a certain role in DOX transmission, these carriers generally have disadvantages such as relatively low drug loading rate and lack of specific recognition. One of the fascinating methods to solve these problems is the utilization of molecularly imprinted polymers (MIPs) such as DDS [12,13]. MIPs are synthetic receptors that can distinctively recognize target molecules in light of the shape, size and chemical function of corresponding binding sites [14,15,16]. Compared with other drug carriers, MIPs have the advantages of selective recognition, great stability, lower cost and uncomplicated synthesis. For example, André Luís MoraisRuela et al. [17] used nicotine and MAA (methacrylate) to synthesize a pH-MIP. The synthesized pH-MIP was subjected to adsorption tested analytes at different pH. The adsorption capacity was obviously higher when pH 6.5 was applied. This was due to the protonation of the nicotine pyrrolidone ring and the carboxylate of the polymer, forming a strong electrostatic force which increased the adsorption of nicotine. At the same time, the author also conducted a comparative release experiment of MIP and none imprinted polymer (NIP) in an aqueous solution of pH 7.4. The results showed that MIP can release 45% of nicotine within 48 h, which was higher than that of NIP (30%), which is a good way to achieve the release of nicotine. Pawley et al. [18] used sensors to depict the drug release behaviors of nanoporous silver organic frameworks and introduced new applications of MIP-based thermal sensing platforms. The silver nanopore matrix applied acetylsalicylic acid (aspirin) as a model drug. The drug release behaviors were investigated by placing the nanomaterials in phosphate buffer for 48 h and determination the drug concentration regularly. Therefore, an acrylamide-based MIP was synthesized, which can detect aspirin in a specific and selective method.

Carbon dots (CDs) is a new groups of carbon materials, which have been proverbially used in many fields. CDs have the advantages of good biocompatibility, low toxicity, nice water solubility, simple functional modification and good chemical inertia [19,20]. The combination of CDs and metal organic framework (MOF) has been widely used. Gu et al. [21] reported a novel bimetallic zirconium hafnium metal organic framework (ZrHf-MOF) inlayed with quantities of CDs (referred as CDs@ZrHf-MOF), which exhibited intense fluorescence and abundant amino functionalization. The prepared CDs@ZrHf-MOF is able to be used as a scaffold to anchor the aptamer chains to mensurate human epidermal growth factor receptor-2 (HER2) and living HER2-overexpressed Michigan Cancer Foundation-7 (MCF-7) cells. The fundamental characterization revealed that these CDs were implanted into the inner cavities of the ZrHf-MOF film without changing the nanostructure, which resulted in nice biocompatibility, intense fluorescence, and eminent electrochemical activity of CDs@ZrHf-MOF. Chen et al. [22] was the first who proposed a simple synthetic route to exploit a new type of MOF-based fluorescence functionalized PCMs (phase change materials) aided by a fluorescence object and a thermal energy guest. Cr-MIL-101-NH_2_ served as a perfect compatible porous carrier, CQD (carbon quantum dot) as an excellent fluorescent active guest and stearic acid as an outstanding thermal energy guest. This distinctive structure can effectively prevent fluorescent quenching induced by conventional aggregation during operation.

**Scheme 1:**
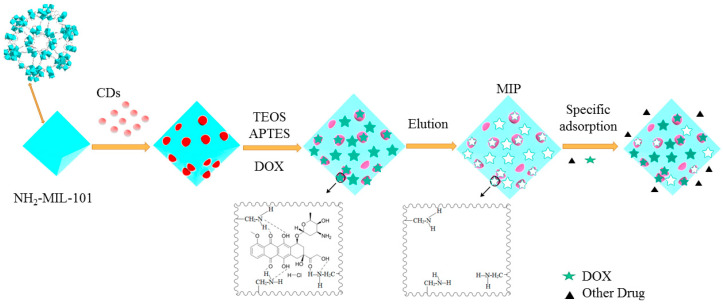
Schematic diagram of the preparation and drug loading of MIP.

Herein, a MOF-based MIP was developed for drug delivery (Scheme 1). MIL and nitrogen-doped CDs (N-CD) were first compounded, and the successfully synthesized material was used as the framework, DOX as template, and tetraethyl orthosilicate (TEOS) as cross-linking agent to synthesize MIP. The as-synthesized N-CDs@MIL-101@MIP nanomaterials were used to sustainable delivery of DOX. Furthermore, the MTT experiment using human hepatocellular liver carcinoma cells (HepG2) and normal human liver cells (HL-7702) were conducted to test the biocompatibility of the synthesized DOX-loaded MIP. As a result, the MIP-synthesized in this study showed an excellent effect in the DDS and may become a potential drug carrier in cancer treatment.

## 2. Materials and Methods

### 2.1. Reagents and Apparatus

Dioscorea opposita Thunb (Chinese yam) was obtained from Jiaozuo Baohetang Pharmacy Co., Ltd. (Jiaozuo, China). Ferric trichloride hexahydrate (FeCl_3_·6H_2_O), 2-aminoterephthalic acid, 3-aminopropyl triethoxysilane (APTES), tetraethoxysilane (TEOS), doxorubicin (DOX), paclitaxel (PTX) and phosphate were purchased from Aladdin Biochemical Technology Co., Ltd (Shanghai, China). The organic solvents such as N,N-Dimethylformamide (DMF), acetate, methanol, ethanol were bought from Tianjin Deen Chemical Reagent Co., Ltd (Tianjin, China). Luteolin (LUT) and ethylenediamine were obtained from Chengdu MUST biotechnology Co., Ltd (Chengdu, China). Acetonitrile was obtained from Merck Drugs & Biotechnology. The reagents used in the cell experiments are all from Zhengzhou Symus Biotechnology Co., Ltd (Zhengzhou, China). The 3-(4,5-dimethythia-zol-2-yl)-2,5-diphenylterazoliumbromide (MTT) was bought from Sigma Aldrich (Shanghai, China) Trading Co., Ltd (Shanghai, China). The HepG2 and HL-7702 cell lines were obtained from the Cell Bank of the Chinese Academy of Sciences (Shanghai, China). All other chemicals were analytical grade.

The shape of nanomaterials was characterized using GeminiSEM500 (Carl Zeiss Inc., Jena, Germany) scanning electron microscope (SEM). An infrared Fourier transform spectrometer (VERTEX 70, Bruker Inc., Karlsruhe, Germany) was used to obtain the FT-IR spectra. The fluorescence performance of the material was measured by a fluorescence spectrometer (F7000, Hitachi Limited., Tokyo, Japan). A TU-1900 spectrophotometer (Beijing Puxi General Instrument Co., Ltd, Beijing, China) was used to obtain the UV-vis absorption of drugs. A microplate reader (CLARIO star, BMG Labtech, Offenburg, Germany) was used for MTT assay.

### 2.2. Preparation of N-Doped CDs (N-CDs)

The N-doped CDs (N-CDs) was typically fabricated by one-pot hydrothermal process [23]. Specifically, 0.5 g powder of yam peel was added into 30 mL deionized water containing 25 μL ethylenediamine. The mixed solution was transferred to a 100-mL volumetric flask and heated at 180 °C for 15 h. The obtained product was centrifuged at 10,000 rpm for 20 min to remove large particles and the supernatant was collected. The as-prepared N-CDs were stored at 4 °C.

### 2.3. Synthesis of N-CDs@MIL-101(Fe) (N-MIL)

MIL was synthesized using hydrothermal method [24]. Briefly, 0.675 g (2.45 mmol) of FeCl_3_·6H_2_O and 0.225 g (1.24 mmol) of 2-aminoterephthalic acid (NH_2_-H_2_BDC) were dissolved in DMF (30 mL). After heating in a Teflon-lined auto-clave at 110 °C for 20 h, the reaction products were re-collected by centrifugation and washed several times with DMF and ethanol, respectively. Finally, the obtained solid was dried at 150 °C for 8 h.

A 0.100-g amount of the above-dried MIL-101 powder was dissolved in 10 mL DMF, 5 mL N-CDs solution was slowly added. After agitating at room temperature for 2 h, the products were collected and washed by ethanol and water for 3 times, respectively. Finally, the acquired solid (N-MIL) was dried at 80 °C for 8 h for following use.

### 2.4. Synthesis of Template MIP Nanoparticles

MIP nanomaterials were synthesized as follows [25]: 0.003 g of DOX as template was dissolved in acetonitrile (3.0 mL). After scattering of 0.050 g N-MIL, APTES (0.600 mL) was introduced into the mixed solution, followed by stirring for 60 min. Afterwards, cross-linker TEOS (0.702 mL) and catalyst (acetic acid, 1.0 mL 0.2 mol/L) were added. After agitating for 30 min, the prepolymerization product was put in a closed container and incubated at 60 °C for 20 h. Then, by 300 mL of methanol/acetic acid, 9:1 *v*/*v*, the obtained MIP was washed to remove the template until DOX was not detected.

### 2.5. Drug Loading Study

DOX adsorbed on the MIP by specific binding sites. One milligram of the MIP or N-MIL was balanced in 3 mL of DOX solution (350 μg/mL) on the shaker oscillator at room temperature for 10 h. Then, the solution was centrifuged, and using UV-vis spectrometry at 480 nm, the adsorption capacity of DOX was determined by observing the absorbance of DOX solution before and after the addition of MIP. The DOX drug loading efficiency (DLE%) of MIP was calculated by the following equation:

(1) $$DLE(\%)=\frac{\mathrm{weight}\;\mathrm{of}\;\mathrm{drug}\;\mathrm{loaded}\;\mathrm{in}\;\mathrm{MIP}}{\mathrm{weight}\;\mathrm{of}\;\mathrm{MIP}\;\mathrm{used}}\times 100$$

For LUT and PTX loading, it is the same procedures as above, just replace the DOX with LUT or PTX.

### 2.6. Release In Vitro

The in vitro drug release research of MIP@DOX was performed in two pH conditions (pH 5.5 and 7.4) at temperature of 37 ± 0.5 °C. In short, 1 mg MIP@DOX was dispersed in the PBS buffer (5 mL) at pH 5.5 or 7.4. These nanomaterials were transferred to the dialysis bag (MWCO 8000–14000, Beijing Solarbio Science & Technology Co., Ltd, Beijing, China), and the dialysis bag was soaked in 100 mL PBS buffer solution with corresponding pH, and vibrated at 37 °C in the dark. At scheduled time intervals, 3 mL of the buffer solution was taking away from the released solution for UV-Vis absorbance determination (480 nm), and an isometric fresh PBS was replenished for following tests. The release process of N-MIL@DOX is the same as MIP@DOX. The cumulative release efficiency was calculated as follows:

(2) $$\mathrm{Cumulative}\;\mathrm{release}\;(\%)\;=\;\frac{C_{n}\times V_{0}+V_{i}\sum_{i=1}^{n-1}C_{i}}{m}\times 100$$

where *C_n_* (µg/mL) represents the concentration of DOX sampling at a specific time. *V*_0_ and *V_i_* (mL) are the total volume of the release medium and the sampling volume, respectively. *m* (mg) is the mass of nanomaterials.

### 2.7. In Vitro Cytotoxicity Test

The vitro cytotoxicity investigations were carried out following the rules of the Declaration of Helsinki of 1975, revised in 2013. According to point 23 of this declaration, before undertaking the research, we have obtained an approval from the Ethics Committee of Biomedical Scientific Research of Henan University (HUSOM2020-037) on 20 March 2020. HepG2 cells (human hepatocellular carcinoma cell line) and HL-7702 (Human normal liver cell line) were maintained in DMEM (Dulbecco’s modified eagle medium) supplemented with 10% (*v*/*v*) FBS (Fetal bovine serum), 100 U·mL^−1^ penicillin and 100 U·mL^−1^ streptomycin under an atmosphere of humidified 5% CO_2_ at 37 °C. Cells were dissociated by trypsinization and then passaged when they reached a density of 106 cell·mL^−1^.

Cell viability was investigated by MTT (3-(4,5-dimethyl-2-thiazolyl)-2,5-diphenyl-2-H-tetrazolium bromide) assay in this work. Specifically, HepG2 or HL-7702 cells in logarithmic growth period were dissociated and seeded to 96-well plates with 2 × 10^4^ cells per well. Then, they were maintained at 5% CO_2_ and 37 °C and for 24 h. Subsequently, 20 μg/mL of N-MIL, N-MIL@DOX, MIP and MIP@DOX were added separately, followed by incubation for 4, 8, 16, 20 and 24 h, respectively. Then, the culture medium was discarded and 1 × PBS was used to wash the cells. Then, 200 μL of MTT (0.5 mg/mL) was introduced and the cells were incubated for 4 h. After that, 100 μL of dimethyl sulfoxide (DMSO) was added to each well after careful removal of supernatants and then the 96-well plate was placed on an oscillator with gentle shaking to make the crystallized formazan sufficiently dissolved in DMSO. Ultimately, the absorbance of each well was detected by a microplate reader at 490 nm.

Interventionary studies involving animals or humans, and other studies requiring ethical approval, must list the authority that provided approval and the corresponding ethical approval code.

## 3. Results and Discussion

### 3.1. Structure Characterization

**Figure 1:**
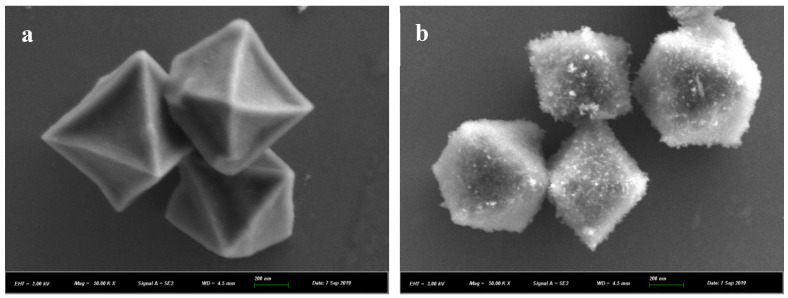

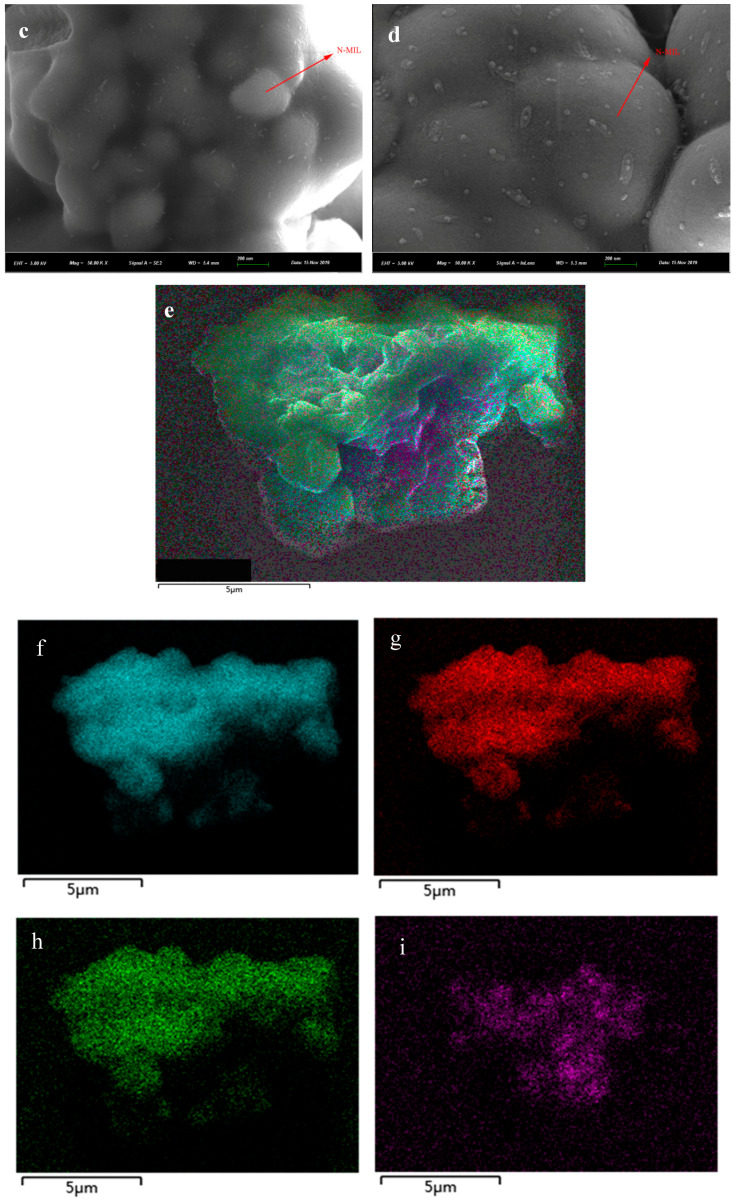
SEM image of MIL (**a**), N-MIL (**b**) and MIP (**c,d**). EDX elemental mapping of MIP (**e**). Blue is O element (**f**), red is C element (**g**), green is N element (**h**), and purple is Fe element (**i**).

The scanning electron microscope (SEM) images of the synthesized MIL and N-MIL were displayed in Figure 1a,b. It was apparent that both MIL and N-MIL nanomaterials had a uniform polyhedron structure. The morphologies of MIL and N-MIL were shown an octahedral structure, though N-MIL was filled with N-CDs. Figure 1c,d were the diagrams of MIP, it was easy to see that MIP was produced by the combination of N-MIL and cross-linking agent. The microstructure and compositional distribution of the MIP nanomaterials were further investigated by EDX (SEM coupled with energy dispersive X-ray) elemental mapping. An overlay of O, C, N and Fe EDX maps was shown in Figure 1e, indicating that Fe mainly located in the core of the material, and its surroundings were cross-linked with N and O, while C can be observed from the surface (Figure 1e). These showed that MIP with N-MIL performance and simple synthesis method was successfully constructed.

**Figure 2:**
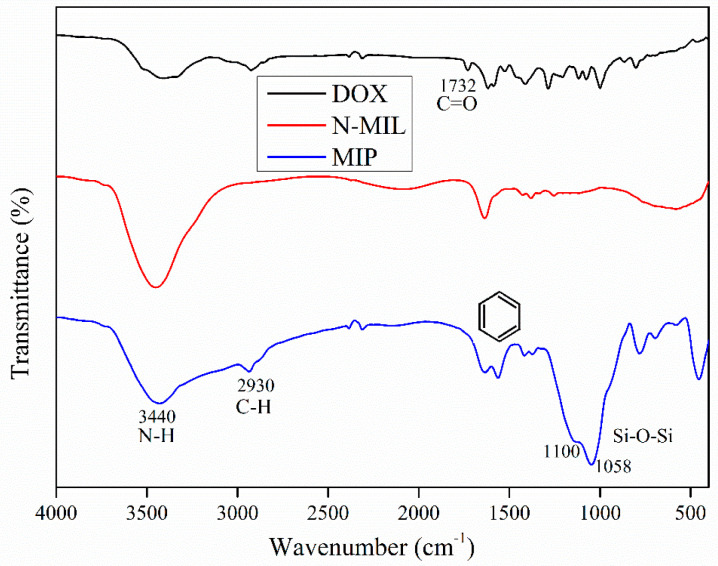
FT-IR spectra of DOX, N-MIL, MIP.

The Fourier transform infrared (FTIR) spectra of synthesized N-MIL, MIP and DOX were recorded in Figure 2. The absorbance peaks at 3430 cm^−1^ was attributed to the stretching vibration and bending vibration of N–H, which was considered to be on NH_2_-MIL, separately [26]. The bands at 2930 cm^−1^ were attributed to the stretching vibration of C–H. The band at 1732 cm^−1^ was the stretching vibration of C=O. The band at 1640–1410 cm^−1^ was a typical band, which was ascribed to the stretching vibration of aromatic benzene rings [27]. Obviously, the appearance of typical bands of the MIP at 1640–1410 cm^−1^ may be attributed to MIL or residual DOX, demonstrating that the imprinted layer was successfully prepared [28]. In addition, the stretching vibrations features of Si–O–Si around 1100 cm^−1^ were observed [29], which indicated that silane coupling agents was hydrolyzed, thus verifying the successful encapsulation process of MIP on MIL-101.

**Figure 3:**
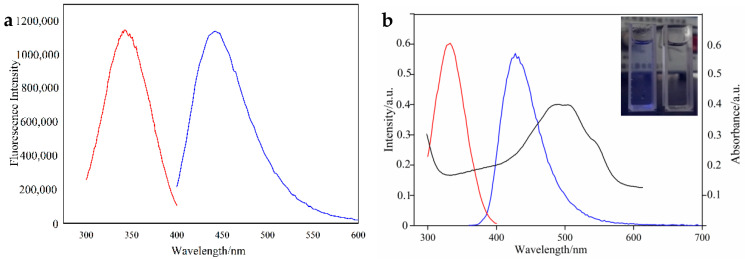
Fluorescence excitation spectrum (red line) and emission spectrum (blue line) of N-MIL (**a**) and MIP (**b**). The black line is the UV-vis absorption spectrum of DOX. Inset: Photographs of MIP under 365 nm UV lamp (left) and natural light (right) irradiation.

The fluorescence performance of the material was measured by a fluorescence spectrometer. As can be seen from Figure 3a, the maximum emission wavelength was 445 nm when the excitation wavelength was 345 nm. As displayed in the inset of Figure 3b, the MIP solution (dissolved in water) was light brown. When irradiated with 365-nm ultraviolet light, it showed bright blue fluorescence. In addition, the fluorescence of MIP come from N-MIL encapsulated inside, and the emission peak position was almost no change compared with that of N-MIL, as shown in Figure 3b. The UV-vis absorption spectrum of DOX had a considerable overlap with the fluorescence emission spectrum of MIP, which indicated that this MIP could serve as a fluorescence donor to generate the energy resonance transfer effect (FRET) with DOX (Figure 3b) [30,31]. The fluorescence characteristics of MIP using DOX as template need to be further exploited for practical application.

### 3.2. Specific Adsorption of MIP on Drugs

**Figure 4:**
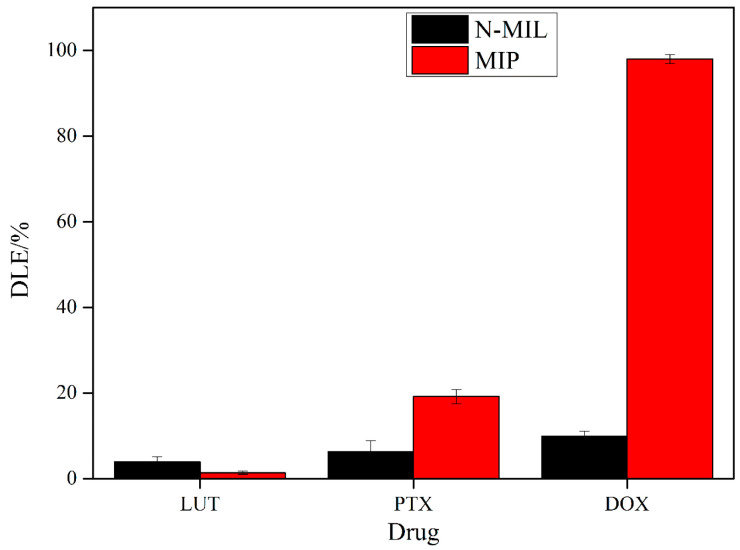
Specificity investigation of MIP. The concentration of LUT, PTX and DOX is 100, 100 and 350 μg/mL, respectively.

The other two drugs (LUT and PTX) that have similar structures as DOX were used to study the specificity of MIP. As shown in Figure 4, comparing with N-MIL, MIP had absolutely specific recognition of DOX. The drug loading rate of MIP to DOX was approximately 100%, while the loading rates of LUT and PTX on MIP were much lower. As showed in Scheme 1, the specificity of MIP to DOX may be due to the hydrogen bonds formed by the amines of the MIP particles (APTES) and the adjacent carbonyl and hydroxyl groups of the DOX. When DOX molecular first entered into the specific cavities of MIP particles, producing inductive fit with the MIP through hydrogen bonds described above. However, comparing with LUT, the higher drug loading rate of PTX on MIP was attributed to its structure being more adjacent carbonyl and hydroxyl groups that can form hydrogen bonds with the MIP. In addition, due to non-specific adsorption, N-MIL has a much lower drug loading capacity for all the three drugs, which also proved that only MIPs had the specificity and sensitivity for drug adsorption. As a result, MIP was synthesized with a certain specificity for the adsorption of DOX in this work.

### 3.3. Drug Loading Studies

**Figure 5:**
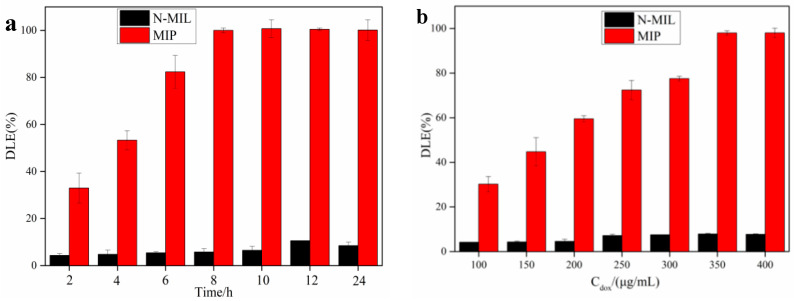
The effect of adsorption time (**a**) and drug concentration (**b**) on DOX loading.

In order to explore the adsorption mechanism, the influence of loading time and DOX concentration on the loading efficiency of MIP were investigated. For comparison purposes, the loading rate of DOX on N-MIL at same conditions was also researched. Totally, as depicted above, due to the specific recognition of MIP on DOX, the drug loading efficiency of MIP was much higher than that of N-MIL at each studied condition. Firstly, the drug loading time of DOX on MIP and N-MIL was studied using 1 mg materials to adsorb 350 μg/mL DOX at room temperature. The results in Figure 5a showed that the adsorption time had significant effect on the drug loading capacity of MIP while the loading time was less than 8 h. When the adsorption time was more than 10 h, the drug loading rate was almost unchanged. Further increasing the loading time, the adsorption rate instead decreased somewhat. We know that there is a balance between adsorption and desorption. After a certain period of time, the adsorption equilibrium reaches, further extending the adsorption time will make the drug that adsorbed on the surface partially desorbed, thus reducing the drug loading efficiency. The loading speed of DOX on MIP/N-MIL was relatively slow and, therefore, the loading process should be accomplished in 10 h.

Moreover, in order to research the effect of DOX concentration on loading efficiency, different concentrations of DOX solution (i.e., 100, 150, 200, 250, 300, 350, and 400 μg/mL) were added using 1 mg MIP. As illustrated in Figure 5b, both drug loading efficiency on MIP and N-MIL increased when the concentration of DOX was increased from 100 to 350 μg/mL and then reached a plateau after 350 μg/mL. It indicated that when the concentration of DOX was 350 μg/mL, the drug loading efficiency of MIP reached to the maximum, which was nearly to 100%, which was much higher than that of N-MIL (7.8%), demonstrating the sensitivity and specificity of MIP to DOX adsorption. We also investigated the relationship between DLE and DOX concentration, and obtained the linear equation as follows: *DLE* = 0.27522*C*_dox_ + 4.02076 (n = 6, r = 0.9938). When the DLE was 50, the concentration of DOX needed was 167 μg/mL, while for N-MIL the maximum DLE was only 7.8%, which also demonstrated the high sensitivity of MIP for DOX loading. The reason for the high DLE of MIP may be that when all the accessible specific cavities of MIP nanoparticles were saturated, the adsorption of analyte was generally attributed to nonspecific interactions, which can be the same for MIP and N-MIL nanoparticles. However, further increasing the concentration of DOX did not change the adsorption efficiency. The results demonstrated that MIP particles possess an identification ability for DOX that can be ascribed to the complementary cavities produced by DOX template. To summarize, the optimal conditions of drug concentration and reaction time were 350 µg/mL and 10 h, respectively. Under the optimum conditions, the maximum amount of DOX adsorbed by MIP was 1029 µg/mg, which accorded with the equilibrium absorption capacity (qe 1250 µg/mg, data not shown). Compared to other reported DDS in Table 1, the drug loading content of the new materials in this work were better than that of the other drug delivery system.

**Figure 6:**
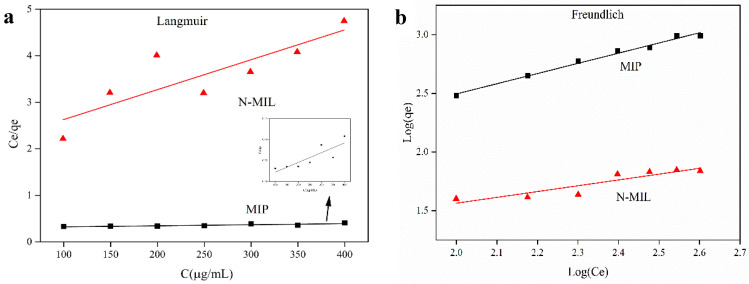
The adsorption isotherms fitted with Langmuir model (**a**) and Freundlich model (**b**).

The results of absorption research were analyzed by Freundlich and Langmuir isotherms to explore the best fitted model to describe the adsorption mechanism (Figure 6a,b), given by Equations (3) and (4) [32,33], respectively:

(3) $$q_{e}=\frac{K_{L}Q_{m}C_{e}}{1+K_{L}C_{e}}$$

(4) $$q_{e}=K_{F}C_{e}{}^{1/n}$$

where *q_e_* is adsorption capacity (mg/g) at equilibrium, *K_L_* is Langmuir constant (L/mg), *q_m_* is maximum monolayer adsorption capacity (mg/g), *K_F_* is Freundlich constant (L/g), and n is Freundlich exponent.

The results of the corresponding parameters acquired by fitting the isothermal models were shown in Table 2. On the whole, a preferable fitting was gained with the Freundlich equation, showing that the adsorption process was non-monomolecular layer chemical adsorption. The DOX molecular may first enter into the specific cavities of MIP particles producing inductive fit with the MIP through hydrogen bonding formed by the amines of the MIP particles (APTES) and the carbonyl groups of the DOX. When all the accessible specific cavities of MIP particles were saturated, the adsorption of analyte was mainly due to nonspecific interactions, which could be the action mode for N-MIL on DOX adsorption.

### 3.4. In Vitro Drug Release

**Figure 7:**
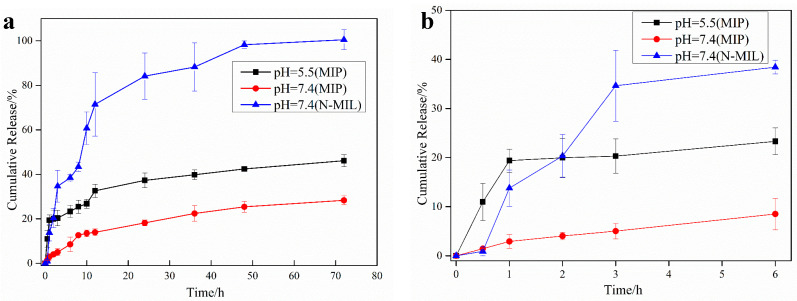
Cumulative percentage of DOX release from N-MIL@DOX and MIP@DOX in PBS (phosphate buffer saline) in 72 h (**a**) and over 6 h (**b**).

Drug release rate analysis was used to evaluate the capability of MIP to deliver DOX, especially through sustainable release at two different pH (5.5, 7.4), so as to simulate the human skin surface and body fluids environment, respectively. The results were compared with drug release from N-MIL particles. Figure 7 showed the pH-responsive cumulative release profiles of MIP@DOX. It can be clearly seen that the drug release of MIP was slower and lower at pH 7.4. The initial rapid release of DOX was probably due to the weak adsorption of DOX molecules on the outer surface of the nanomaterials. It was noteworthy that N-MIL nanomaterials released a large quantity of DOX within 48 h (approximately 98% of DOX at pH 7.4), while MIP only released 28.03% of drug within 72 h at pH 7.4. Even at pH 5.5, the cumulative release from MIP was eventually 46.19% after 72 h. Consequently, although MIP and N-MIL both have sustained-release effects, the release of DOX from MIP nanomaterials was much slower and more delayed. The significant differences in the release of DOX from MIP nanoparticles and N-MIL nanoparticles can be owing to the presence of a specific site in the imprinted nanoparticles that strongly interacted with DOX molecules.

There are different models that have been applied to describe drug release kinetics from porous carriers, such as Korsmeyer–Peppas (Equation (5)) [34] and Sigmoidal model (Equation (6)) [35].

(5) $$Q=Kt^{n}$$

(6) $$Q=\frac{R_{s}}{1+e^{-k_{s}(t-t_{50})}}$$

where *Q* represents the cumulative release percentage at time *t* (h), *K* is the kinetic constant, *n* is diffusion exponent. The theoretical maximum release rate is *R_s_* (%), *k_s_* and *t*_50_ are the release kinetic constant.

The results of the kinetic parameters for each model were summed up in Table 3. There was a noticeable distinction in the release kinetics of different nanomaterials under these conditions. Sigmoidal model cannot fit the experimental data well for MIP, while the Korsmeyer–Peppas model was more suitable for data fitting. However, for N-MIL system at pH 7.4, the experimental data did not conform well to the Korsmeyer–Peppas model, but the data had a good fit with the Sigmoidal model. According to the fitting results of Korsmeyer-Peppas in MIP sustained-release system in Table 3, n was 0.26 at pH 5.5 which was, less than 0.45, revealing that the release kinetics process of the sustained-release system conformed to Fick diffusion mechanism. That was the interaction between MIP and drug was relatively weak. When pH was 7.4, it was 0.45 < n < 0.89, demonstrating that the release kinetics of this system conformed to the non-Fick diffusion mechanism, and the interaction between MIP and the drug was obviously enhanced. Under different pH conditions, the sustained-release mechanism of MIP system changed, which was mainly due to the partial protonation of MIP at pH 5.5, which weakened the hydrogen bonding force between amino groups of MIP cavity and carbonyl groups of DOX. When pH was 7.4, the system was neutral, and the force between MIP and DOX increased. On the contrary, for the N-MIL release system, it was quickly released within the first 12 h, and then released in an S shape until it reached the platform. The abnormal non-Fick spread is described by Sigmoidal release function. The change of drug diffusion and internal stresses resulted the abnormalities. The relaxation and diffusion time scales of macromolecular are similar in anomalous non-Fick diffusion [36,37,38,39,40].

### 3.5. Cytotoxicity Test

**Figure 8:**
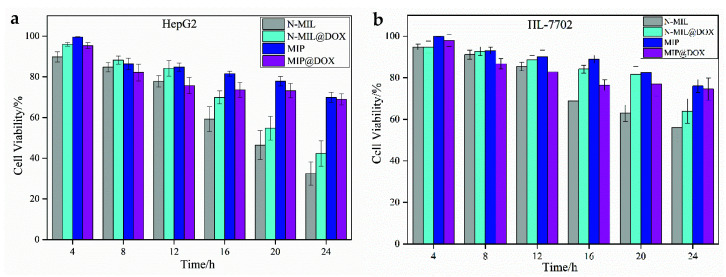
Cell viability of HepG2 cells (**a**) and HL-7702 cells (**b**) treated with N-MIL, N-MIL@DOX, MIP and MIP@DOX at different time.

To evaluate the potential application of the constructed MIP nanoparticles in DDS, the cytotoxicity tests of MIP and N-MIL on HepG2 cells and HL-7702 cells [41,42,43,44,45,46] were detected by MTT method (Figure 8). The cell viability showed that the inhibition rate increased with time increasing. Within 20 h, the inhibition rates of MIP on HL-7702 cells and HepG2 cells were lower than 20%, showing that MIP had no significant toxicity. Even after 24 h, the rate of cell survival can still reach 76% and 70%. However, after 24 h of incubation with the same concentration as MIP (20 μg/mL), MIP@DOX inhibited HepG2 and HL-7702 cells by 31% and 25%, respectively, which were higher than that of MIP. The better inhibition rate of MIP@DOX on HepG2 was due to the slow drug release of DOX in this system. N-MIL and N-MIL@DOX systems also had the ability to inhibit HepG2 cells, but they can also kill normal liver HL-7702 cells at the same time. These results indicated that the toxicity of MIP to living cells was low in a certain time, and prove that the synthesized MIP has low toxicity and good biocompatibility.

To sum up, we synthesized a MIP with high drug loading efficiency, low toxicity and high biological activity. Before it is formally used as a clinical drug carrier, there may still be some restrictions for its application, such as the lack of in vivo experimental data support. Due to the poor water solubility of the synthesized MIP, which may be caused by the large size, it is not conducive to animal experiments. Therefore, our following work is to synthesize small-sized and water-soluble nanomaterials that can be clinically used for drug carriers. At present, this work is in progress.

## 4. Conclusions

In summary, we compounded MIL and N-CDs, and then successfully used the nanocomposite materials as framework, and DOX as template to synthesize MIP. Adsorption experiments showed that MIP had high sensitivity, specific selectivity and superior efficiency for DOX loading. It has a sustained release effect for DOX which cumulative release rates in 72 h were 46.19% and 28.03% at pH 5.5 and 7.4, respectively. The cytotoxicity test results indicated that the inhibit rate of MIP on HepG2 and HL-7702 were 30.1% and 23.93%, respectively, showing its good biological activity. In view of the outstanding biocompatibility, superior drug-loading and sustained releasing properties, this molecular imprinted polymer may have a potential to be used as a promising carrier in slow-released drug system in the future.

## Figures and Tables

**Table 1:** Comparison of the drug loading content of N-MIL/MIP with other reported drug delivery systems.

Author	Drug Delivery System	Drug Loading Rate	Reference
Jiang Lan	Amphiphilic polyrotaxane-based block copolymer	25.5%	[11]
Peng Huhong	Chitosan nanoparticle	90%	[27]
Wang Haiyan	Fluorescent double template imprinted polymer	11.05%	[28]
Shi Yuqiong	N-MIL-MIP	97.9%	This work

**Table 2:** Isotherm parameters for DOX adsorption by MIP nanoparticles.

	Langmuir			Freundlich		
	*q*_m_ (mg/g)	*K*_L_ (L/mg)	*R* ^2^	*K*_F_ (L/mg)	1/*n*	*R* ^2^
**N-MIL**	140.85	0.00332	0.7316	2.9060	0.5571	0.8415
**MIP**	5000	0.000669	0.7523	5.7227	0.8688	0.9877

**Table 3:** Correlation coefficient (R^2^) of the release mechanism model Sigmoidal and Korsmeyer–Peppas according to various pH.

Condition		Korsmeyer–Peppas			Sigmoidal Model			
		K	n	R^2^	k_s_	R_s_	t_50_	R^2^
MIP	pH = 5.5	15.7	0.26	0.95	0.18	41.7	4.89	0.85
	pH = 7.4	2.63	0.59	0.97	0.20	24.1	9.55	0.91
N-MIL	pH = 7.4	7.89	0.26	0.76	0.27	93.1	7.68	0.95
